# Insulin resistance indicators in aortic disease: A large cohort study

**DOI:** 10.1002/ctm2.70570

**Published:** 2026-01-07

**Authors:** Zehua Shao, Zhongyuan Lu, Wanxin Duan, Lingchen Huang, Yangxue Sun, Lixi Gan, Yue Gu, Hongwei Guo

**Affiliations:** ^1^ Department of Surgery, Fuwai Hospital, National Center for Cardiovascular Diseases Chinese Academy of Medical Sciences and Peking Union Medical College Beijing China; ^2^ Department of Anesthesiology and Perioperative Medicine People's Hospital of Zhengzhou University Henan Provincial People's Hospital Zhengzhou Henan Province China; ^3^ Department of Pulmonary and Critical Care Medicine Zhongshan Hospital Fudan University Shanghai Medical College Shanghai China; ^4^ Department of Nephrology Henan Clinical Medical Research Center for Nephropathy Henan Provincial Key Laboratory of Kidney Disease and Immunology Henan Provincial People's Hospital Zhengzhou University People's Hospital Henan University People's Hospital Zhengzhou Henan China

**Keywords:** aortic aneurysm, aortic dissection, Mendelian randomisation, triglyceride‐glucose index, UK Biobank

## Abstract

**Background:**

The triglyceride‐glucose index (TyG), a novel marker of insulin resistance, is recognised as a risk factor for multiple cardiovascular diseases. The link between its risk and aortic dissection and aortic aneurysm (AD/AA) is not well‐defined. This research seeks to explore the relationship.

**Methods:**

This study analysed 386 063 participants from the UK Biobank, a large prospective cohort, all initially free of AD/AA. The main focus was on the occurrence rate of AD/AA. Multivariate Cox regression models were used to analyse the association between the TyG index, its related parameters, and the risk of AD/AA. Association was further validated using a real‐world clinical cohort from Central China Fuwai Hospital. A two‐sample Mendelian randomisation (MR) analysis utilising the inverse variance weighting method was conducted to investigate the causal link between TyG and AD/AA.

**Result:**

Among the 386 063 participants in the UK Biobank cohort, 3805 cases of AD/AA were reported. After controlling for covariates, a higher TyG index and its related parameters were associated with an increased incidence of AD/AA. The hazard ratios (HRs) were as follows: TyG (HR = 1.14, 95% CI: 1.07–1.22, *p* <.001), TyG‐WHR (HR = 1.17, 95% CI: 1.12–1.22, *p* <.001), and TyG‐WHtR (HR = 1.11, 95% CI: 1.06–1.16, *p* <.001). Association between TyG and the risk of aortic diseases was also replicated in the single‐centre cohort. Two‐sample MR analysis indicated strong evidence of a causal relationship between genetically predicted TyG levels and AA (OR = 1.97, 95% CI: 1.37–2.84, *p* <.001), while no significant association was observed with AD (OR = 0.79, 95% CI: 0.31–1.99, *p* = .61).

**Conclusion:**

Through complementary epidemiological, clinical, and genetic approaches, our findings indicate that elevated TyG index represents a robust and potentially causal risk factor for aortic diseases (especially AA). These results highlight the importance of metabolic risk assessment in aortic disease prevention and emphasise the need for further mechanistic studies to understand the differential links between TyG and specific aortic phenotypes.

**Highlights:**

- Higher TyG index and related ratios are linked to increased AD/AA risk.
- Prospective cohort analysis confirms robust linear associations.
- Mendelian randomisation supports a causal role of TyG in AA.
- Findings highlight TyG as a potential target for early prevention.

## Graphical abstract

Elevated TyG index and its related parameters were associated with an increased risk of aortic diseases in a prospective cohort of 386 063 participants. Mendelian randomisation suggested a potential causal link with AA but not with aortic dissection (AD).

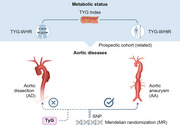

## 1 INTRODUCTION

Aortic diseases, such as aortic dissection (AD) and aneurysms (AA), pose significant cardiovascular risks due to their high mortality rates upon rupture or complications.^1^, ^2^ An aneurysm is characterised by a localised, irreversible dilation affecting all vessel wall layers, leading to a vessel diameter increase of at least 1.5 times. Aortic aneurysms can increase the risk of aortic dissection by progressively dilating the artery, ultimately leading to destabilisation of the aortic wall. In the United States, aortic dissection occurs in 5–30 individuals per million each year, with mortality reaching up to 50% without prompt treatment.^3^ The mortality rate for ruptured aortic aneurysms can be as high as 90%.^4^ Both conditions are closely linked to aging, smoking, hypertension, dyslipidemia, and connective tissue disorders, including Marfan syndrome, Loeys–Dietz syndrome, and Ehlers–Danlos syndrome.^5^, ^6^, ^7^, ^8^ Given their insidious onset, diagnostic challenges, and potentially fatal outcomes, an in‐depth analysis of AD and AA risk factors is crucial. Such knowledge could guide clinicians in developing effective management strategies and support the implementation of targeted public health policies for high‐risk populations. Recently, increasing attention has been paid to the role of metabolic disorders in the development of AD/AA.^9^, ^10^, ^11^ The hyperinsulinemic‐euglycemic clamp, although the gold standard for assessing insulin sensitivity, is impractical for large‐scale screening or routine clinical use due to its time‐consuming, costly, and invasive nature.^11^ The triglyceride‐glucose (TyG) index has been identified as an efficient, affordable, and reliable indicator of insulin resistance (IR).^12^, ^13^

Insulin resistance physiologically hinders the suppression of hepatic gluconeogenesis and the clearance of triglycerides, leading to increased fasting blood glucose and triglyceride levels. The TyG index, reflecting dual metabolic disturbances, is computed using the formula: ln [TG (mg/dL) × FBG (mg/dL)/2].^12^ It has shown a strong correlation with the hyperinsulinemic‐euglycemic clamp and HOMA‐IR, confirming its effectiveness in various populations.^14^, ^15^ Given these advantages, the TyG index is widely adopted in epidemiological studies to assess metabolic risk and insulin resistance burden.

Besides TyG, anthropometric measures like waist circumference (WC), hip circumference (HC), waist‐to‐hip ratio (WHR), and waist‐to‐height ratio (WHtR) serve as important indicators of metabolic status.^16^ Recent studies have proposed TyG‐derived composite indices (e.g., TyG‐WHR and TyG‐WHtR) as improved predictors of cardiometabolic risk.^17^ While the TyG index and related parameters are linked to various cardiovascular diseases, their specific association with AD/AA is not well understood and requires further study to establish causal relationships

Mendelian randomisation (MR) utilises genetic variations, such as single nucleotide polymorphisms (SNPs), as instrumental variables to robustly estimate the causal effects of risk factors on disease outcomes.^18^ MR utilises genetically determined variants that are randomly assigned, reducing residual confounding and reverse causation typical in observational studies, thereby elucidating the causal link between exposure and outcome.^19^ Thus, MR offers a viable method for evaluating the causal link between the TyG index and AD or AA.

This study investigated the link between TyG and its related parameters with AD/AA risk using UK Biobank cohort data. Our findings were validated through rigorous adjustment for confounding variables and sensitivity analyses, confirming the robustness of the observed associations. Moreover, we employed a two‐sample MR approach to strengthen the causal inference of this association.

## 2 METHODS

### 2.1 Study design overview and population

The research was conducted in two primary phases. Initially, we thoroughly analysed the association between the TyG index, its related indices, and AD/AA, considering various potential confounders, using UK Biobank data. In the second phase, summary statistics from genome‐wide association studies (GWAS) of TyG and AD/AA were extracted for two‐sample MR analysis.

**FIGURE 1:**
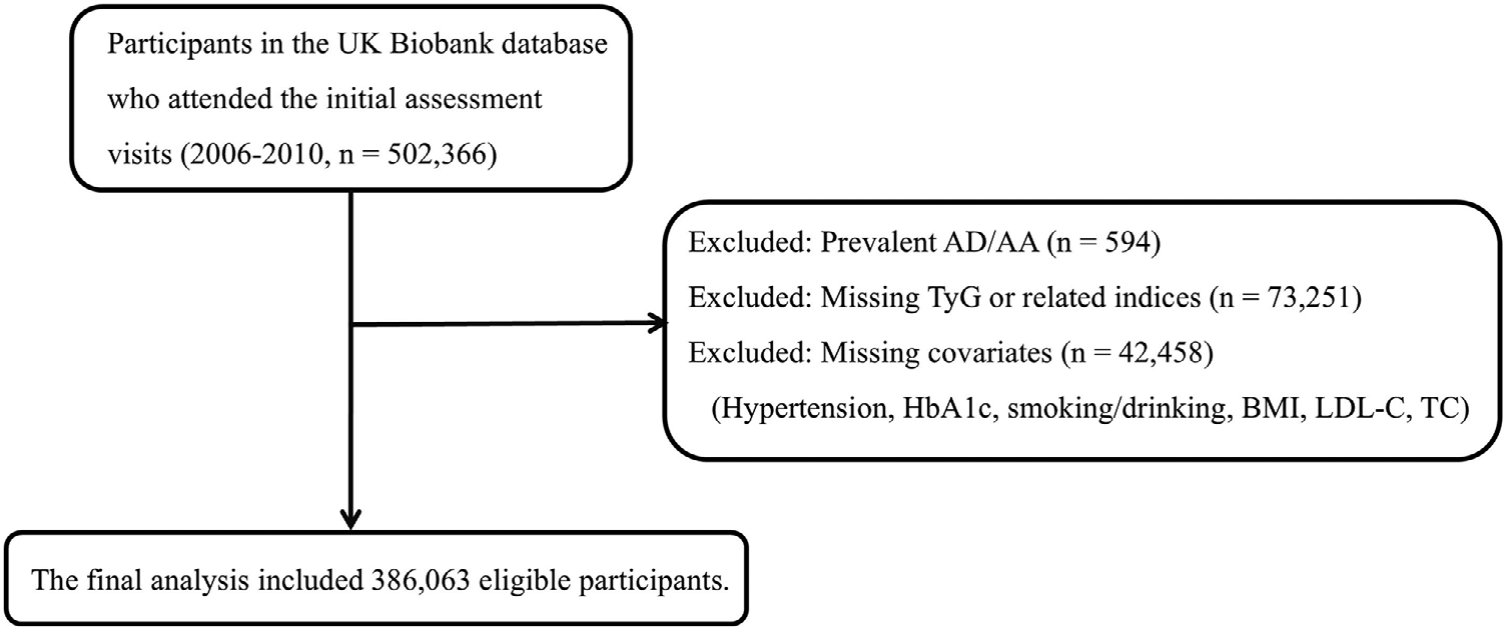
Flowchart of study participant selection and inclusion criteria.

The UK Biobank is a large‐scale, prospective, community‐based cohort study aimed at advancing biomedical research and informing public health policy. Between 2006 and 2010, 502 357 volunteers aged 40 to 69 were recruited. This dataset comprises demographic details, health and lifestyle factors, physical examination outcomes, functional assessments, and biological samples such as blood, urine, and saliva. Data collection and research within the UK Biobank complied with rigorous ethical and privacy standards, obtaining written informed consent from all participants before enrolment. The Northwest Multicentre Research Ethics Committee approved the study, which adheres to the Declaration of Helsinki principles. Participants diagnosed with AD/AA (*n* = 585), connective tissue disease (*n* = 8329), or those lacking TG (*n* = 33 233) and FBG (*n* = 39 918) data were excluded from the analysis. The final analytical sample included 386 063 eligible participants. A detailed breakdown of the inclusion process is presented in the flowchart (Figure 1).

For detailed information on all GWAS data sources, please refer to Table S1. The SNPs used in the MR study were derived from several large GWAS studies involving non‐overlapping populations. The TyG index was derived from the UK Biobank cohort, utilising TyG‐related genetic variants identified in prior GWAS. Previous studies on the GWAS for TyG involved 273 368 participants aged 40–69 years, excluding those with diabetes or lipid metabolism disorders.^20^, ^21^, ^22^, ^23^ Summary data for aortic dissection (AD) with 881 cases and 349 539 controls, diagnosed using ICD‐10 codes I71.00‐I71.03, and abdominal aortic aneurysm (AA) with 7395 cases and 349 539 controls, diagnosed using ICD‐10 codes I71.1‐171.6, I71.8, I71.9, were sourced from the FinnGen Consortium^24^ (https://www.finngen.fi/en).

### 2.2 Data collection and definitions

The study used data from the UK Biobank repository. The covariates considered were age (Field 21022), sex (Field 31), ethnicity (self‐reported at baseline, Field 21000), height (Field 50), weight (Field 21002), BMI (Field 21001), waist circumference (Field 48), hip circumference (Field 49), systolic blood pressure (Field 4080), diastolic blood pressure (Field 4079), smoking status (Field 20116), alcohol consumption (Field 30750), triglycerides (Field 30870), HDL cholesterol (Field 30760), LDL cholesterol (Field 30780), and total cholesterol (Field 30690). These variables were selected because they have been consistently identified as established risk factors for aortic diseases in epidemiological studies.^25^, ^26^ Diabetes mellitus was characterised by a blood glucose level of ≥7.0 mmol/L or HbA1c >6.5%, in conjunction with a self‐reported diagnosis. Hypertension was defined as SBP ≥140 mmHg or DBP ≥ 90 mmHg, in addition to self‐reported diagnosis. Smoking status was categorised as ‘yes’ or ‘no’, while alcohol consumption was recorded as ‘current drinker’, ‘former drinker’, or ‘non‐drinker’. WHR = WC/HC; WHtR = WC/height. The TyG index is determined using the formula: Ln[triglycerides (mg/dL) × glucose (mg/dL) / 2]. Additionally, TyG‐WHR is calculated as TyG multiplied by WHR, and TyG‐WHtR as TyG multiplied by WHtR.

### 2.3 Outcome

The diagnosis of AD/AA was determined using the ICD‐10 classification, with AD assigned code I71.0 and AA assigned codes I71.1–I71.9, as documented in the UK Biobank hospitalisation records. Participants were monitored from recruitment until the earliest of a first recorded AD/AA event or death. The follow‐up period concluded upon the occurrence of either event.

### 2.4 Statistical analysis

In the UK Biobank cohort, TyG was computed as a continuous variable to assess its relationship with AD/AA. The Kolmogorov–Smirnov test was used to evaluate normality. Continuous variables with non‐normal distributions were reported as medians and interquartile ranges (IQR), whereas categorical variables were expressed as counts and percentages. Chi‐square tests assessed differences in categorical variables between AD/AA and non‐AD/AA groups, and Mann–Whitney *U* tests compared continuous variables with non‐normal distributions.

Cox proportional hazards models were used to evaluate the relationship between the TyG index (and its related indices) and the risk of AD/AA across three multivariate regression models. Model 1 did not include any variable adjustments, whereas Model 2 was adjusted for age, gender, race, and BMI. Model 3 included further adjustments for smoking, alcohol consumption, HbA1c, hypertension, cholesterol, and LDL, in addition to age, sex, race, and BMI. Notably, BMI was not adjusted for in Models 2 and 3 when examining associations between TyG‐WHR/WHtR and AD/AA risk, as these variables already accounted for BMI‐related factors.

To evaluate the robustness and consistency of our findings, we conducted sensitivity analyses. Hazard ratios (HRs) and 95% confidence intervals (CIs) were calculated using Fine–Gray competing risk regression models, accounting for all‐cause mortality as a competing risk. We performed subgroup analyses considering age (<60 vs. ≥60), sex (female vs. male), smoking status (yes vs. no), alcohol consumption (never, previous, current), diabetes mellitus (yes vs. no), hypertension (yes vs. no), and BMI categories (underweight, normal weight, overweight, obese).

### 2.5 Two‐sample MR analysis

The MR approach is based on three fundamental assumptions to evaluate the causal link between an exposure and an outcome: (1) There must be a robust association with the exposure. (2) They should not be influenced by any confounders related to the exposure‐outcome relationship. (3) They must affect the outcome (aortic dissection/aortic aneurysm) exclusively through the exposure and not via alternative pathways.

The SNPs linked to the TyG index have been identified in prior research and are well‐established.^20^, ^21^, ^22^, ^23^ The GWAS involved 273 368 participants aged 40–69 years, excluding those with diabetes or lipid metabolism disorders. SNPs linked to triglycerides, glucose, and other factors such as systolic and diastolic blood pressure and BMI were excluded due to linkage disequilibrium (*R* ^2^ < 0.01, kb = 10 Mb) to mitigate horizontal pleiotropy (*p* < 5 × 10^−^⁸). A total of 192 SNPs were chosen as instrumental variables for the TyG index (Table S2).

The primary statistical method used to aggregate causal estimates across SNPs was the random‐effects inverse variance weighted (IVW) approach. While IVW method provides robust results when all three MR assumptions are satisfied, it is sensitive to horizontal pleiotropy. To address this, MR‐Egger regression and the weighted median method were applied as complementary analyses. These methods are based on different assumptions, and consistent results across all approaches would strengthen the robustness of our findings. Cochran's Q test was employed to evaluate SNP heterogeneity, considering *p* <.05 as indicative of significant heterogeneity. MR‐Egger regression assessed bias from horizontal pleiotropy, using the intercept as an indicator, where *p* <.05 indicates pleiotropic bias. The overall estimate's stability was evaluated through a ‘leave‐one‐out’ analysis, sequentially removing each SNP to assess its impact on the result. Additionally, funnel plots were generated for visual inspection of asymmetry and further sensitivity testing.

To enhance the causal inference between genetically predicted TyG index and aortic disease, we conducted a two‐sample MR analysis using two independent GWAS datasets for AD (GCST90436158, https://doi.org/10.1038/s41588‐018‐0184‐y) and AA (GCST90480203, https://doi.org/10.1126/science.adj1182). The same set of TyG‐associated SNPs (after LD clumping and pleiotropy filtering) were retained as instrumental variables to ensure methodological consistency. MR analyses employed IVW as the main method, supplemented by MR‐Egger, weighted median, heterogeneity testing, horizontal pleiotropy assessment (MR‐Egger intercept), and the MR‐PRESSO global test. Comprehensive results are available in Table S3 and Figure S2.

**FIGURE 2:**
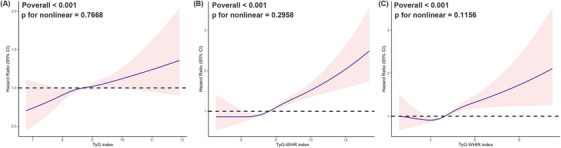
Restricted cubic spline (RCS) curves for the association between TyG‐related indices and AD/AA risk. RCS curves showing the adjusted association between TyG‐related indices and the risk of aortic dissection and aneurysm (AD/AA). Models were adjusted for age, sex, smoking, drinking, BMI, hypertension, LDL‐c, and HbA1c. Solid lines indicate hazard ratios; shaded areas represent 95% confidence intervals. (A) TyG in AD/AA, (B) TyG‐WHR in AD/AA, (C) TyG‐WHtR in AD/AA.

Data cleaning and statistical analyses were conducted using R software (version 4.3.3). A *p*‐value less than.05 in a two‐tailed test was deemed statistically significant.

### 2.6 Single‐centre observational validation cohort

To further strengthen the robustness of our findings, we performed an external validation using a real‐world clinical cohort from Central China Fuwai Hospital. We retrospectively included all patients who were admitted between 1 January, 2024 and 1 November, 2025. Individuals diagnosed with aortic diseases (including aortic aneurysm or aortic dissection) constituted the case group, while health‐exam participants without cardiovascular disease served as the control group. A total of 1736 patients with AD/AA and 18 779 controls were included.

Fasting triglycerides and glucose levels were extracted from admission laboratory tests to calculate the TyG index. The association between the TyG index and the presence of AD/AA was assessed using logistic regression models. Six models were developed: one unadjusted and five adjusted for variables including age, sex, blood pressure, LDL‐C, total cholesterol, BMI, and key metabolic factors. Results are expressed as odds ratios (ORs) accompanied by 95% confidence intervals (CIs).

## 3 RESULTS

### 3.1 Basic characteristics of participants

Table 1 presents the baseline characteristics of participants categorised by AD/AA status. The cohort's median age was 58 years, with an interquartile range of 50 to 63 years. Compared to individuals in non‐AD/AA group, those in the AD/AA group exhibited higher values for age, weight, height, BMI, triglycerides, SBP, DBP, glucose, HbA1c, WC and HC. The AD/AA group included a higher percentage of male, white, and smoking participants. In contrast, this group showed lower levels of LDL‐c and HDL‐c and a smaller proportion of never smokers. The AD/AA group exhibited significantly higher TyG levels than the non‐AD/AA group (*p* <.001). Prior to modelling the associations between TyG‐related indicators and aortic disease risk, we assessed potential non‐linear relationships through restricted cubic spline regressions. The results revealed an approximately linear dose–response trend across the range of TyG, TyG‐WHR, and TyG‐WHtR values, supporting the use of these variables as continuous terms in subsequent multivariable models (Figure 2).

**TABLE 1:** Baseline characteristics of participants.

Characteristics	Overall *N* = 386 063	Without AA/AD *N* = 382 258	AA/AD *N* = 3805	*p* Value
Age, years	58.00 (50.00, 63.00)	58.00 (50.00, 63.00)	63.00 (59.00, 67.00)	<.001
**Sex**				
Female	207 325 (53.70)	206 508 (54.02)	817 (21.47)	<.001
Male	178 738 (46.30)	175 750 (45.98)	2988 (78.53)	
**Race**				
White	350 773 (90.86)	347 212 (90.83)	3561 (93.59)	<.001
Others	35 290 (9.14)	35 046 (9.17)	244 (6.41)	
Weight, kg	76.60 (66.60, 87.80)	76.60 (66.60, 87.70)	83.70 (73.80, 94.30)	<.001
Height, cm	168.00 (162.00, 175.00)	168.00 (162.00, 175.00)	173.00 (167.00, 179.00)	<.001
BMI, kg/m^2^	26.78 (24.17, 29.94)	26.77 (24.16, 29.93)	27.78 (25.20, 30.84)	<.001
LDL‐c, mmol/L	3.52 (2.94, 4.12)	3.52 (2.95, 4.12)	3.38 (2.75, 4.05)	<.001
HDL‐c, mmol/L	1.40 (1.17, 1.67)	1.40 (1.17, 1.68)	1.22 (1.03, 1.46)	<.001
TC, mmol/L	5.65 (4.91, 6.42)	5.65 (4.91, 6.43)	5.39 (4.57, 6.26)	<.001
TG, mmol/L	1.49 (1.05, 2.16)	1.49 (1.05, 2.15)	1.72 (1.22, 2.48)	<.001
SBP, mmHg	138.00 (126.00, 152.00)	138.00 (126.00, 152.00)	144.00 (131.00, 158.00)	<.001
DBP, mmHg	82.00 (75.00, 89.00)	82.00 (75.00, 89.00)	84.00 (77.00, 92.00)	<.001
**Hypertension**				
No	142 738 (36.97)	142 268 (37.22)	470 (12.35)	<.001
Yes	243 325 (63.03)	239 990 (62.78)	3335 (87.65)	
Glucose, mmol/L	4.93 (4.60, 5.32)	4.93 (4.60, 5.32)	4.96 (4.61, 5.37)	.019
HbA1c, %	5.38 (5.15, 5.63)	5.37 (5.15, 5.62)	5.47 (5.24, 5.74)	<.001
FBG, mmol/L	3.00 (2.00, 4.00)	3.00 (2.00, 4.00)	3.00 (3.00, 4.00)	<.001
**Diabetes**				
No	364 902 (94.52)	361 353 (94.53)	3549 (93.27)	.001
Yes	21 161 (5.48)	20 905 (5.47)	256 (6.73)	
**Smoking**				
No	154 165 (39.93)	153 296 (40.10)	869 (22.84)	<.001
Yes	231 898 (60.07)	228 962 (59.90)	2936 (77.16)	
**Drinking**				
Never	17 244 (4.47)	17 125 (4.48)	119 (3.13)	<.001
Previous	13 775 (3.57)	13 577 (3.55)	198 (5.20)	
Current	355 044 (91.96)	351 556 (91.97)	3488 (91.67)	
Waist circumference, cm	90.00 (81.00, 99.00)	90.00 (81.00, 99.00)	97.00 (89.00, 105.00)	<.001
Hip circumference, cm	102.00 (97.00, 108.00)	102.00 (97.00, 108.00)	103.00 (99.00, 109.00)	<.001
TyG index	8.69 (8.32, 9.08)	8.69 (8.32, 9.08)	8.84 (8.48, 9.23)	<.001

Abbreviations: AA, aortic aneurysm; AD, aortic dissection; BMI, body mass index; DBP, diastolic blood pressure; FBG, fasting blood glucose; HbA1c, glycated haemoglobin; HDL‐c, high‐density lipoprotein cholesterol; LDL‐c, low‐density lipoprotein; SBP, systolic blood pressure; TC, total cholesterol; TG, triglyceride; TyG, triglyceride‐glucose.

### 3.2 The relationships between TyG, TyG‐WHR, TyG‐WHtR, and the risk of AD/AA

Over a median follow‐up period of 13.7 years, 3805 AD/AA cases were documented. Multivariate Cox regression analysis indicated that elevated TyG, TyG‐WHR, and TyG‐WHtR levels were linked to a higher relative risk of AD/AA development in the unadjusted model (*p* <.001 for all trends; Table 2). Specifically, each standard deviation (SD) increase in these metrics was associated with a 54% higher risk of AD/AA occurrence (HR = 1.54, 95% CI: 1.46–1.63) for TyG, a 64% increase (HR = 1.64, 95% CI: 1.60–1.69) for TyG‐WHR, and a 49% increase (HR = 1.49, 95% CI: 1.44–1.55) for TyG‐WHtR. These associations persisted in Model 2 even after controlling for age, sex, race, and BMI. In Model 3, which included additional covariates, each standard deviation increase in the metrics corresponded to a 14% higher risk for TyG (HR = 1.14, 95% CI 1.07–1.22), a 17% increase for TyG‐WHR (HR = 1.17, 95% CI 1.12–1.22), and an 11% increase for TyG‐WHtR (HR = 1.11, 95% CI 1.06–1.16) (Table 2). In the sensitivity analysis, the results remained significant when using the Fine–Gray competing risk regression models, which accounted for all‐cause mortality competing risks. A one standard deviation increase in the TyG metric correlated with a 14% higher risk of AD/AA incidence (HR = 1.14, 95% CI 1.07–1.22). An increase of one standard deviation in the TyG‐WHR metric corresponded to a 17% elevated risk of AD/AA incidence (HR = 1.17, 95% CI 1.13–1.22). An increase of one standard deviation in the TyG‐WHtR metric is associated with an 11% higher risk of AD/AA onset (HR = 1.11, 95% CI 1.06–1.16) (Table 2). These findings validate the strong link between TyG‐related indices and AD/AA risk.

**TABLE 2:** Baseline TyG index and incident risks of AD/AA.

	TyG		TyG‐WHR		TyG‐WHtR	
	HR (95% CI)	*p* Value	HR (95% CI)	*p* Value	HR (95% CI)	*p* Value
**Cox proportional hazards model**						
Model 1	1.54 (1.46, 1.63)	<.001	1.64 (1.60, 1.69)	<.001	1.49 (1.44, 1.55)	<.001
Model 2	1.11 (1.05, 1.18)	.001	1.22 (1.18, 1.26)	<.001	1.21 (1.16, 1.26)	<.001
Model 3	1.14 (1.07, 1.22)	<.001	1.17 (1.12, 1.22)	<.001	1.11 (1.06, 1.16)	<.001
**Fine–Gray competing risk regression model**						
Sensitivity analysis	1.14 (1.07, 1.22)	<.001	1.17 (1.13, 1.22)	<.001	1.11 (1.06, 1.16)	<.001

*Note*: Model 1: unadjusted model; Model 2: adjusted for age, gender, race, BMI; Model 3: adjusted for age, gender, race, BMI, smoking status, drinking status, hypertension, LDL‐c, HbA1c, TC. Sensitivity analysis: adjusted for age, gender, race, BMI, smoking status, drinking status, hypertension, LDL‐c, HbA1c, TC.

BMI was not adjusted for in Models 2 and 3 when examining associations between TyG‐WHR/WHtR and AD/AA risk, as these variables already accounted for BMI‐related factors.

### 3.3 Subgroup analysis

**FIGURE 3:**
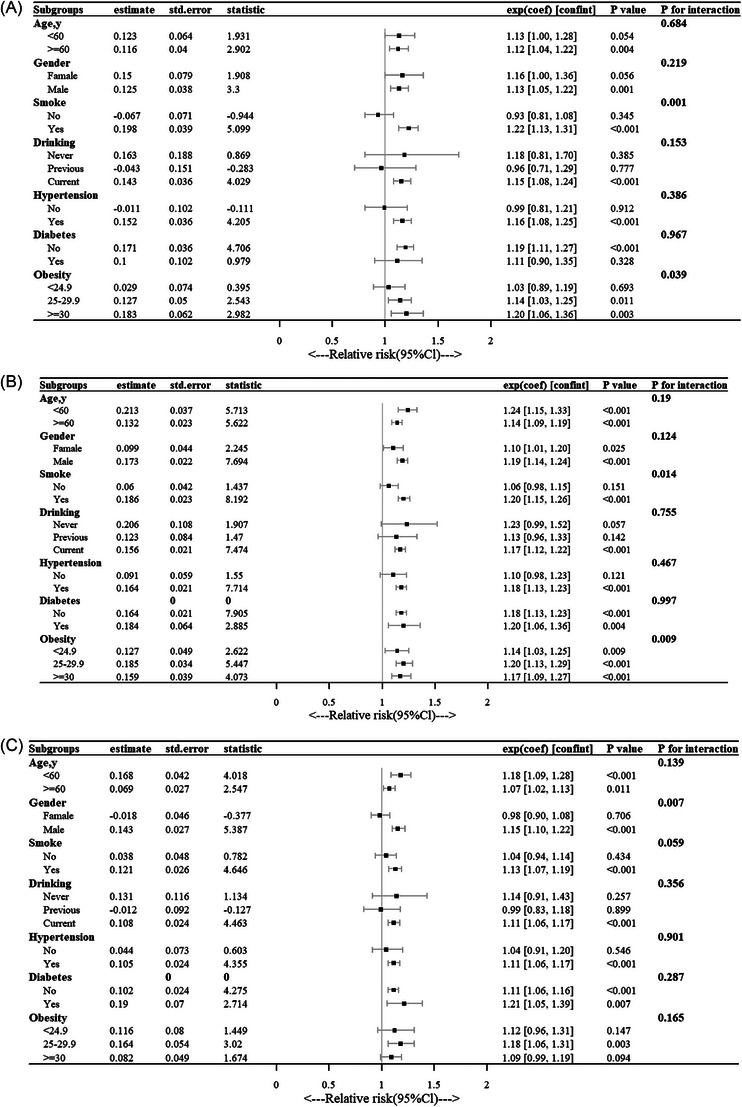
Subgroup analyses examining the correlation between TyG and its associated parameters with the risk of AD/AA and its subtypes. (A) TyG‐AD/AA, (B) TyG WHR‐ AD/AA, (C) Tyg WHtR‐ AD/AA.

Figure 3 illustrates subgroup analyses on the correlation between TyG, its parameters, and the risk of AD/AA and its subtypes. A significant interaction was observed between smoking status and the TyG index (*p* <.001 for interaction), with the association being stronger in smokers than in nonsmokers (HR = 1.22, 95% CI: 1.13–1.31 vs. HR = 0.93, 95% CI: 0.81–1.08). Obesity significantly interacted with the TyG index (*p* <.001), with a stronger correlation between TyG and AD/AA risk in obese individuals compared to those with normal or overweight status (HR = 1.20, 95% CI 1.06–1.36 vs. HR = 1.03, 95% CI 0.89–1.19 vs. HR = 1.14, 95% CI 1.03–1.25). Comparable patterns were observed for TyG‐WHR in relation to AD/AA risk. Stratified analyses indicated a sex‐based interaction with TyG‐WHtR, showing a stronger association with AD/AA risk in males (HR = 1.15, 95% CI 1.10–1.22) compared to females (HR = 0.98, 95% CI 0.90–1.08).

### 3.4 A two‐sample Mendelian analysis was conducted to investigate the relationship between TyG and AD/AA

We performed a one‐way two‐sample MR analysis to investigate the causal link between the TyG index and the risk of AD and AA, as shown in Table 3. The study found that a higher TyG index significantly increased the risk of AA (OR = 1.97, 95% CI 1.37–2.84, *p* <.001), but did not significantly affect the risk of AD (OR = 0.79, 95% CI 0.31–1.99, *p* = .61). Additional methods, such as MR‐Egger and weighted median approaches, corroborated our primary findings. Despite observing heterogeneity (Cochran's *Q* = 221.57, *p* <.01 for AA; *Q* = 181.94, *p* = .07 for AD), the MR‐Egger intercept showed no evidence of horizontal pleiotropy for the selected SNPs (intercept = –0.0054, *p* = .15 for AA; intercept = 0.0108, *p* = .25 for AD) for the selected SNPs. The results of the sensitivity analysis are detailed in Table S3.

**TABLE 3:** MR estimates from each method of assessing the causal effect of TyG on the risk of AD/AA.

Exposure	Outcome	MR method	Number of SNPs	OR	SE	95% confidence interval	*p* Value
**TyG**	**AD**	IVW	156	1.219	0.287	0.694–2.139	4.91E‐01
		MR Egger	156	0.787	0.474	0.311–1.992	6.14E‐01
		Weighted median	156	1.201	0.431	0.516–2.795	6.71E‐01
**TyG**	**AA**	IVW	153	1.590	0.114	1.272–1.989	4.79E‐05
		MR Egger	153	1.971	0.187	1.366–2.844	3.95E‐04
		Weighted median	153	1.908	0.169	1.371–2.655	1.28E‐04

A ‘leave‐one‐out’ sensitivity analysis was conducted for the TyG index to verify that no individual SNP had an undue impact on the findings. The analysis verified that the causal impact of TyG on AD/AA was consistent, with no notable variations linked to any specific SNP. Furthermore, the funnel plot analysis revealed a symmetrical distribution of the results, suggesting the absence of significant bias. The detailed information is shown in Figure S1.

By leveraging two independent GWAS datasets, the direction and magnitude of the causal estimates remained highly consistent with our primary MR findings. The genetically predicted TyG index demonstrated a strong causal link with AA (IVW OR = 1.89, 95% CI: 1.03–2.77; MR‐Egger OR = 1.97; Weighted median OR = 1.91), while no significant causal relationship was found for AD (IVW OR = 1.89, 95% CI: 0.67–2.14; *p* = .49). MR‐Egger intercepts were nonsignificant for both outcomes (*p* >.05), indicating no evidence of directional pleiotropy. The MR‐PRESSO global test indicated no significant distortion for AA (*p* = .596), while AD showed significant distortion (*p* <.001) with no outliers detected post‐correction. Leave‐one‐out and funnel plots further demonstrated the stability and symmetry of SNP‐specific estimates (Figure S2).

### 3.5 Single‐centre cohort validation

In the independent single‐centre cohort, the TyG index consistently correlated with aortic diseases (see Table S4). Logistic regression analyses consistently indicated that elevated TyG levels were linked to higher odds of AD/AA in all models. In the unadjusted model, TyG was significantly associated with an increased risk of AD/AA (OR = 1.325, 95% CI: 1.236–1.421, *p* = 2.49 × 10^−15^). The association remained significant after adjusting for age and sex (OR = 1.087, 95% CI: 1.001–1.179, *p* = .047). Further adjustment for blood pressure strengthened the association (OR = 1.140, 95% CI: 1.051–1.237, *p* = .0016).

Models additionally adjusting for LDL‐C (OR = 1.199, *p* = 5.75×10^−^⁶), total cholesterol (OR = 1.660, *P* < 2×10^−^16), or lipid principal components (OR = 1.401, *p* = 1.11×10^−^⁵) further supported the robustness of this association. No evidence of multicollinearity was detected across models (all VIF < 5).

## 4 DISCUSSION

This extensive prospective cohort study examined the relationship between the TyG index, along with its related indices (TyG‐WHR and TyG‐WHtR), and the incidence of AD/AA. After adjusting for demographic, lifestyle, and metabolic factors, elevated TyG, TyG‐WHR, and TyG‐WHtR levels were associated with a higher risk of AD/AA. MR analysis results indicated a causal relationship between TyG and AA risk, but not with AD. To enhance the robustness of causal inference, we conducted a second two‐sample MR analysis utilising independent outcome datasets from the GWAS Catalog. Consistent with our primary findings, TyG showed a stable causal effect on AA but not on AD, supporting the reliability of our results across different genetic sources. As MR analyses rely on the random assortment of genetic variants to reduce residual confounding and reverse causation, these findings strengthen the argument that TyG may contribute directly to the pathogenesis of AA.

Using real‐world data from 1736 AD/AA cases and 18 779 controls, we observed a consistent and directionally concordant association between higher TyG levels and the presence of aortic diseases, even after extensive adjustments for age, sex, blood pressure, lipid profiles, and major metabolic factors. The persistence of these associations across multiple models, combined with the absence of multicollinearity, provides strong external support for the epidemiological relationship observed in the UK Biobank and the causal inference derived from MR analysis. This triangulation of evidence across population‐based, genetic, and hospital‐based datasets substantially enhances confidence in the role of TyG as a metabolic indicator relevant to aortic disease risk.

The TyG index has been well‐studied in the context of coronary artery disease, ischemic stroke, and other macrovascular complications. Several observational studies have also reported a link between TyG and thoracic or abdominal aortic aneurysms.^27^, ^28^ Additionally, numerous studies have demonstrated that Tyg‐related indices may exhibit both linear and nonlinear associations with cardiovascular disease risk.^29^, ^30^, ^31^, ^32^ After adjusting for demographic, lifestyle, and metabolic factors, we found that elevated TyG, TyG‐WHR, and TyG‐WHtR were each associated with an increased risk of AD/AA, aligning with previous reports. Our study stands out due to its large cohort size, comprehensive multivariable adjustments, and the use of two‐sample MR for causal inference, overcoming limitations of previous research with smaller samples or cross‐sectional designs. The observed divergence in the MR results for AD and AA highlights the heterogeneous nature of aortic diseases and the need for condition‐specific research.

From a mechanistic standpoint, these findings are in line with emerging evidence that chronic metabolic disturbances – especially IR – may predispose the aortic wall to pathological remodelling. We consider that chronic IR can initiate or exacerbate several processes implicated in aortic pathology. Initially, insulin resistance (IR) is linked to disrupted nitric oxide signalling, increased oxidative stress, and elevated pro‐inflammatory cytokines, which can gradually weaken the aortic wall's structural integrity.^9^, ^10^, ^11^ Second, persistent low‐grade inflammation, a hallmark of metabolic syndrome, promotes the macrophage infiltration and other immune cells into the arterial wall, thereby increasing matrix metalloproteinase (MMP) activity. Heightened MMP activity weakens the extracellular matrix, fostering aneurysm formation and expansion.^9^ Third, prolonged exposure to dysregulated lipid profiles and hyperglycemia could enhance smooth muscle cell apoptosis and fibroelastic tissue degradation, leading to aortic dilation.

Our results suggest thatchronic inflammatory and metabolic derangements collectively predispose individuals to AA formation, consistent with the stronger causal inference observed in our MR analysis for AA. Aneurysms typically develop over a protracted period, and the long‐term effects of IR‐related stress may be central to its pathophysiology.^33^, ^34^ Conversely, the absence of strong causal evidence between TyG and the development of AD in our genetic study implies that AD may be driven by other factors. Unlike AA, which is characterised by gradual vessel dilation, AD often arises from a sudden tear in the aortic intima superimposed on chronic degenerative changes. In some patients, acute haemodynamic stress (e.g., abrupt blood pressure surges) may overshadow the contribution of metabolic dysfunction to initiating an intimal tear.^35^, ^36^ Besides, genetic predispositions (e.g., connective tissue disorders) and acute triggers (e.g., uncontrolled hypertension) may have a more dominant role in AD than in AA, thereby diluting the detectable causal impact of IR in MR analyses.^37^, ^38^, ^39^ It remains plausible that metabolic factors contribute to AD risk through more complex or indirect mechanisms not fully captured by the genetic instruments used in our MR study. Larger, more specific genetic datasets focusing on AD subtypes (e.g., Stanford Type A vs. Type B) and incorporating more detailed phenotyping may yield additional insights.^40^, ^41^

Subgroup analysis revealed that the TyG index more effectively predicts the risk of AD/AA in smokers and individuals with obesity. These subgroups demonstrated heightened sensitivity to TyG index variations, suggesting that targeted monitoring in these groups could substantially mitigate AD/AA risk. Notably, current smokers and obese individuals exhibited steeper risk gradients. The synergistic effect observed in smokers likely reflects the compounding influence of tobacco‐induced systemic inflammation and oxidative stress, which amplifies insulin resistance‐driven inflammatory pathways, accelerates atherosclerosis, and exacerbates the adverse impacts of elevated TyG levels.^42^, ^43^ Smoking is known to promotes inflammatory cells infiltration into vascular walls and accelerates extracellular matrix degradation.^43^, ^44^ Similarly, the obesity's synergistic effect may be attributed to adipose tissue endocrine dysregulation. Previous research indicates that visceral fat worsens vascular endothelial dysfunction in insulin‐resistant conditions by releasing free fatty acids and pro‐inflammatory agents, promoting arterial damage.^45^

Our findings also offer potential clinical and public health implications. The TyG index, derived from standard fasting blood glucose and triglyceride tests, offers a straightforward and economical assessment tool for both primary care and specialised medical environments. Incorporating TyG and anthropometrically adjusted parameters into standardised cardiovascular risk screening protocols may enable earlier identification of high‐risk individuals and promote further diagnostic evaluations, such as imaging‐based assessments or specialised follow‐up of aortic pathology. Moreover, targeted interventions aimed at mitigating IR, such as exercise programs, nutritional counselling, and possibly pharmacologic agents with insulin‐sensitising properties, might confer protective effects in patients with elevated TyG levels. Although these implications are promising, prospective trials are needed to determine whether lowering the TyG index leads to a measurable decline in aortic disease incidence or progression.

Our findings indicate a positive link between insulin resistance, measured by the TyG index, and the risk of aortic diseases. However, prior retrospective studies have documented a negative correlation between diabetes and aortic aneurysm or dissection.^46^, ^47^ This discrepancy may be attributed to survival bias and residual confounding in earlier studies, where diabetic patients might die of other cardiovascular conditions before developing aortic events.^48^ Our prospective design and Mendelian randomisation analysis reduce these biases and offer more robust causal inference. Moreover, diabetes is not equivalent to insulin resistance. The TyG index captures subclinical metabolic dysfunction that may precede a clinical diagnosis of diabetes and thus may serve as a more sensitive indicator of early pathophysiological changes associated with aortic disease.

In interpreting the divergent findings between the observational and MR analyses, it is important to consider the distinct pathophysiological mechanisms of AD and AA. AD is typically an acute event characterised by intimal tear and medial disruption, often precipitated by hypertension and shear stress,^49^, ^50^ whereas AA represents a chronic process involving extracellular matrix degradation, vascular inflammation, and smooth muscle cell apoptosis.^51^ These differences may explain the observed causal association between TyG and AA, but not AD, in the MR analysis. Since insulin resistance contributes to low‐grade inflammation, oxidative stress, and matrix remodelling, it may have a greater impact on aneurysm formation than dissection.^52^ The TyG index could be a more sensitive indicator of chronic metabolic damage linked to AA, but its relevance in the acute pathology of AD might be restricted.

Emerging evidence suggests multiple pathophysiological mechanisms through which insulin resistance, as reflected by an elevated TyG index, may contribute to aortic wall degeneration and the development of aortic aneurysm and dissection. First, insulin resistance leads to compensatory hyperinsulinemia, thereby exacerbating vascular inflammation.^53^ Secondly, insulin resistance has been linked to heightened activity of MMPs, especially MMP‐2 and MMP‐9, which break down elastin and collagen in the aortic extracellular matrix, resulting in structural weakening and increased risk of dilation.^54^

Moreover, oxidative stress induced by metabolic dysfunction contributes to endothelial dysfunction and vascular smooth muscle cell (VSMC) apoptosis, further compromising aortic integrity.^55^, ^56^ Insulin resistance may also impair vascular repair and remodelling processes, disrupting the balance between matrix synthesis and degradation, and promoting fibrosis and stiffness in the arterial wall.^57^, ^58^, ^59^ These mechanisms collectively offer a credible biological connection between a high TyG index and a heightened risk of aortic diseases.

It is important to recognise certain limitations. First, despite controlling for many potential confounders, residual confounding cannot be fully excluded given the observational nature of the study. Second, the predominance of individuals of European ancestry in the UK Biobank limits the applicability of our findings to other ethnic and racial groups. Third, the use of hospital registry data for the diagnosis of AD/AA may introduce misclassification bias or underdiagnosis, and the relatively lower incidence of AD compared to AA could affect statistical power in certain analyses. Fourth, while our MR design helps address confounding and reverse causation, the potential influence of horizontal pleiotropy (in which genetic variants influence outcomes through multiple pathways) remains a source of potential bias.^40^, ^41^ Fifth, the limited number of AD cases in this cohort leads to reduced statistical power and unstable estimates in stratified analysis. Consequently, we did not perform distinct analyses for AD and AA in the cohort study, which might have restricted our capacity to identify condition‐specific associations between the TyG index and either AD or AA. Finally, a more detailed phenotyping that distinguishes different subtypes of AD and AA may yield additional clarity about the precise mechanisms by which metabolic disturbances affect aortic integrity.^34^, ^35^

## 5 CONCLUSION

This large‐scale prospective cohort study provides both epidemiological and genetic evidence that higher TyG and TyG‐related indices are associated with an increased risk of aortic diseases. MR analyses further support a possible causal role of TyG in the development of AA. These observations indicate the importance of managing metabolic risk factors – particularly IR – in the prevention of AA, while highlighting the need for further investigation into the distinct pathophysiological and genetic determinants of AD. Future research focusing on targeted preventive strategies and mechanistic trials will be invaluable for determining whether lowering TyG can effectively modify the trajectory of aortic disease and improve patient outcomes.

## AUTHOR CONTRIBUTIONS

*Conceptualisation*: Zehua Shao and Zhongyuan Lu. *Methodology*: Zehua Shao; Zhongyuan Lu and Lingchen Huang. *Software*: Yue Gu and Zhongyuan Lu. *Validation*: Yangxue Sun and Lixi Gan. *Formal Analysis*: Zehua Shao and Zhongyuan Lu. *Investigation*: Zehua Shao and Zhongyuan Lu. *Resources*: Yangxue Sun and Lixi Gan. *Data Curation*: Yue Gu and Lixi Gan. *Writing – Original Draft*: Zehua Shao and Zhongyuan Lu. *Writing – Review & Editing*: Wanxin Duan; Hongwei Guo and Yue Gu. *Visualisation*: Zhongyuan Lu and Zehua Shao. *Supervision*: Hongwei Guo and Wanxin Duan. *Project Administration*: Zehua Shao and Hongwei Guo.

## ETHICS STATEMENT

The hospital‐based case–control cohort from Central China Fuwai Hospital was conducted in accordance with institutional ethical standards and the Declaration of Helsinki. Ethical approval for this study was obtained from the Ethics Committee of Central China Fuwai Hospital (Approval 2025. No. 85). The requirement for informed consent was waived by the committee because the study used de‐identified clinical and imaging data with no direct patient contact. The protocols involving human participants received ethical approval from the UK Biobank and the North West Multi‐Center Research Ethics Committee (REC reference: 11/NW/0382). Written informed consent was obtained from all participants before their involvement in the study.

## Supporting information



Supporting Information

Supporting Information

Supporting Information

## Data Availability

The hospital‐based dataset from Central China Fuwai Hospital contains sensitive clinical information and is therefore not publicly available. Access to the de‐identified data may be granted upon reasonable request and with approval from the Central China Fuwai Hospital Ethics Committee (Approval 2025. No. 85). The remaining original contributions of the study are included in the article/supplementary material, and further inquiries should be directed to the corresponding author.
