# Supplementary material for: Insulin resistance indicators in aortic disease: A large cohort study
Source: Clin Transl Med. 2026 Jan 7;16(1):e70570. doi: 10.1002/ctm2.70570 (PMC12778938; doi:10.1002/ctm2.70570)
Supplement: Supplementary file 1 — Supporting Information [file CTM2-16-e70570-s002.docx]

**Supplementary Table 1. Summary of GWAS included in this study**

| **Year** | **Trait** | **Population** | **Cases** | **Controls** | **Samplesize** | **Websource** |
| --- | --- | --- | --- | --- | --- | --- |
| 2020 | TyG | European | NA | NA | 273,368 | DOI: 10.3389/fcvm.2020.583473 |
| 2023 | Aortic dissection | European | 881 | 34,9539 | 320,404 | www.finngen.fi/en |
| 2023 | Aortic aneurysm | European | 7395 | 349,539 | 33,925 | www.finngen.fi/en |
| 2018 | Aortic dissection | European | NA | NA | 408,961 | GWAS Catalog: [GCST90436158](https://www.ebi.ac.uk/gwas/studies/GCST90436158)  DOI:[10.1038/s41588-018-0184-y](https://doi.org/10.1038/s41588-018-0184-y) |
| 2018 | Aortic aneurysm | European | NA | NA | 635,969 | GWAS Catalog: GCST90480203 DOI: 10.1126/science.adj1182 |

**Supplementary Table 2.** SNPs used as genetic instruments for this study on TyG index

| **SNP** | **REF** | **ALT** | **BETA** | **SE** | **P** |
| --- | --- | --- | --- | --- | --- |
| rs114165349 | C | G | 0.0445298 | 0.00465259 | 1.07e-21 |
| rs72904790 | C | T | -0.0137147 | 0.00240632 | 1.2e-08 |
| rs213498 | A | T | -0.00802754 | 0.00146386 | 4.17e-08 |
| rs10889332 | T | C | -0.0388967 | 0.00143046 | 1.34e-162 |
| rs72669514 | T | C | 0.018609 | 0.00320686 | 6.53e-09 |
| rs17656269 | T | C | 0.00874937 | 0.00147002 | 2.65e-09 |
| rs16836630 | C | G | -0.0173377 | 0.00252122 | 6.14e-12 |
| rs1760801 | A | G | -0.00895698 | 0.0015125 | 3.19e-09 |
| rs340836 | C | T | -0.00869811 | 0.00139646 | 4.71e-10 |
| rs76172548 | C | A | 0.0228139 | 0.00383242 | 2.64e-09 |
| rs3120619 | A | G | 0.0118809 | 0.0018033 | 4.45e-11 |
| rs11118610 | C | A | -0.00902819 | 0.00138913 | 8.09e-11 |
| rs4846922 | T | C | 0.0221545 | 0.00146286 | 8.63e-52 |
| rs907866 | A | G | -0.00927654 | 0.00138756 | 2.31e-11 |
| rs111585158 | T | C | 0.0121133 | 0.00211397 | 1e-08 |
| rs144470864 | C | A | 0.0172612 | 0.00307793 | 2.05e-08 |
| rs76384951 | C | A | -0.0295509 | 0.0025237 | 1.16e-31 |
| rs533617 | C | T | -0.0464535 | 0.00346743 | 6.48e-41 |
| rs35750610 | C | T | 0.0185128 | 0.00234709 | 3.09e-15 |
| rs34921778 | G | A | 0.00842606 | 0.00144997 | 6.21e-09 |
| rs12617848 | T | C | 0.0142794 | 0.00200585 | 1.09e-12 |
| rs80216311 | T | C | -0.0141686 | 0.00238957 | 3.05e-09 |
| rs61737373 | A | G | -0.028607 | 0.00292878 | 1.57e-22 |
| rs6547692 | G | A | 0.0375073 | 0.00138448 | 2.05e-161 |
| rs10206462 | C | T | -0.00893744 | 0.00142769 | 3.85e-10 |
| rs6760053 | G | C | -0.00778921 | 0.00137713 | 1.55e-08 |
| rs6710938 | C | A | -0.00898973 | 0.00161797 | 2.76e-08 |
| rs79953491 | G | A | -0.0237048 | 0.00210804 | 2.49e-29 |
| rs115128825 | A | C | 0.026901 | 0.00487631 | 3.46e-08 |
| rs484066 | A | T | -0.0159198 | 0.00141892 | 3.31e-29 |
| rs17694506 | C | T | 0.00900471 | 0.00141859 | 2.19e-10 |
| rs2943645 | C | T | -0.0209209 | 0.0014316 | 2.4e-48 |
| rs6437249 | T | C | 0.00838469 | 0.00149284 | 1.95e-08 |
| rs147764624 | C | G | -0.03019 | 0.00550582 | 4.18e-08 |
| rs390802 | A | G | -0.0153354 | 0.00176462 | 3.63e-18 |
| rs62271373 | A | T | 0.0253586 | 0.00303878 | 7.15e-17 |
| rs13074711 | C | T | 0.0120107 | 0.00218488 | 3.86e-08 |
| rs13108218 | A | G | 0.0156395 | 0.00143815 | 1.54e-27 |
| rs71603401 | G | A | 0.0125089 | 0.00205483 | 1.15e-09 |
| rs6448429 | T | C | 0.0126753 | 0.00187808 | 1.49e-11 |
| rs1471251 | T | A | 0.0164471 | 0.00140761 | 1.56e-31 |
| rs4134363 | A | G | -0.00950005 | 0.00170166 | 2.37e-08 |
| rs3822076 | A | T | 0.00867089 | 0.0013818 | 3.5e-10 |
| rs2035816 | G | A | -0.0159447 | 0.00249077 | 1.54e-10 |
| rs78025076 | T | C | 0.0271124 | 0.00482777 | 1.96e-08 |
| rs390556 | C | T | -0.0132704 | 0.00220378 | 1.73e-09 |
| rs72754154 | A | G | -0.0213999 | 0.00314107 | 9.58e-12 |
| rs3936511 | G | A | 0.0216126 | 0.00174659 | 3.69e-35 |
| rs151913 | A | G | 0.00786984 | 0.00141384 | 2.6e-08 |
| rs7703744 | G | C | -0.0109345 | 0.00155172 | 1.84e-12 |
| rs72801474 | A | G | -0.0146422 | 0.00235286 | 4.88e-10 |
| rs12173130 | C | T | 0.00971075 | 0.00176945 | 4.07e-08 |
| rs11134475 | A | G | -0.0169417 | 0.00142319 | 1.15e-32 |
| rs2963476 | G | A | 0.0133691 | 0.00169886 | 3.58e-15 |
| rs6923241 | T | C | -0.0109234 | 0.00154721 | 1.67e-12 |
| rs2745400 | A | G | 0.00826032 | 0.00137239 | 1.76e-09 |
| rs2894211 | A | C | 0.0174652 | 0.00218851 | 1.46e-15 |
| rs7758790 | C | T | 0.0142803 | 0.00164795 | 4.52e-18 |
| rs55697600 | G | A | 0.0351072 | 0.00360432 | 2.05e-22 |
| rs185139895 | A | G | 0.0207564 | 0.00338431 | 8.63e-10 |
| rs3025053 | A | G | -0.0134581 | 0.00212481 | 2.4e-10 |
| rs4715317 | T | G | 0.00974329 | 0.00143947 | 1.3e-11 |
| rs1967685 | C | G | -0.0142769 | 0.0013714 | 2.25e-25 |
| rs632057 | T | G | 0.0153665 | 0.00142077 | 2.94e-27 |
| rs12208357 | T | C | 0.0219558 | 0.00272455 | 7.75e-16 |
| rs77009508 | G | A | 0.0224178 | 0.00259593 | 5.86e-18 |
| rs55730499 | T | C | -0.0182481 | 0.0025524 | 8.74e-13 |
| rs186696265 | T | C | -0.0472632 | 0.00592501 | 1.51e-15 |
| rs4709746 | T | C | -0.0112466 | 0.00203182 | 3.11e-08 |
| rs852424 | T | C | 0.00852818 | 0.0014628 | 5.55e-09 |
| rs38205 | A | C | 0.00791466 | 0.00143983 | 3.87e-08 |
| rs2106727 | A | G | -0.0108194 | 0.00142823 | 3.59e-14 |
| rs4722551 | C | T | -0.0185803 | 0.00188091 | 5.21e-23 |
| rs1534696 | C | A | 0.0107368 | 0.00137638 | 6.18e-15 |
| rs2971676 | A | G | 0.0133494 | 0.00239848 | 2.61e-08 |
| rs878521 | A | G | 0.0217619 | 0.00158662 | 8.43e-43 |
| rs62459110 | C | G | -0.0213924 | 0.00364332 | 4.32e-09 |
| rs799157 | T | C | 0.0407906 | 0.00340184 | 4.05e-33 |
| rs17145750 | T | C | -0.0560614 | 0.00185642 | 5.28e-200 |
| rs10260148 | T | C | 0.0149589 | 0.00154174 | 2.96e-22 |
| rs73198299 | C | T | 0.0122709 | 0.00222876 | 3.68e-08 |
| rs7821812 | C | G | 0.0163357 | 0.00169741 | 6.39e-22 |
| rs904009 | C | A | 0.0159306 | 0.00162585 | 1.16e-22 |
| rs4921914 | C | T | 0.0194386 | 0.00165914 | 1.07e-31 |
| rs2975424 | C | T | 0.0106046 | 0.0017554 | 1.53e-09 |
| rs1388941 | A | G | 0.0143536 | 0.00145941 | 8.01e-23 |
| rs268 | G | A | 0.10865 | 0.00515243 | 1.25e-98 |
| rs117026536 | T | G | -0.0951759 | 0.00226332 | 1e-200 |
| rs57295072 | C | G | -0.0304863 | 0.00469748 | 8.6e-11 |
| rs17091881 | C | T | 0.0785949 | 0.00427426 | 1.82e-75 |
| rs74444445 | C | T | 0.034928 | 0.00488632 | 8.82e-13 |
| rs117805502 | T | C | -0.0321654 | 0.00439412 | 2.48e-13 |
| rs28550053 | G | A | -0.0177064 | 0.00182104 | 2.42e-22 |
| rs75662196 | C | G | -0.0279294 | 0.00434519 | 1.3e-10 |
| rs17092008 | T | C | 0.0208253 | 0.00285324 | 2.91e-13 |
| rs11781356 | A | T | 0.00993171 | 0.00176553 | 1.85e-08 |
| rs2081687 | T | C | 0.011677 | 0.00145382 | 9.63e-16 |
| rs71525127 | G | C | 0.019603 | 0.00254745 | 1.42e-14 |
| rs11558471 | G | A | -0.011475 | 0.00147145 | 6.29e-15 |
| rs17321515 | G | A | -0.0439 | 0.00137141 | 1e-200 |
| rs62521590 | G | T | 0.0146537 | 0.00155763 | 5.11e-21 |
| rs10811661 | C | T | -0.00987338 | 0.0018054 | 4.53e-08 |
| rs13289566 | T | C | -0.0118284 | 0.00166888 | 1.37e-12 |
| rs2244278 | A | C | -0.0133902 | 0.00211876 | 2.62e-10 |
| rs3750571 | A | C | -0.0124087 | 0.00189812 | 6.27e-11 |
| rs11006681 | A | G | -0.0110061 | 0.00184368 | 2.38e-09 |
| rs142164605 | A | T | -0.0177614 | 0.00278129 | 1.71e-10 |
| rs10786069 | C | T | 0.0130977 | 0.00137832 | 2.06e-21 |
| rs113344423 | A | G | 0.0212993 | 0.00301795 | 1.7e-12 |
| rs2792736 | T | A | -0.0100505 | 0.00153811 | 6.4e-11 |
| rs10832027 | G | A | -0.0122569 | 0.00148245 | 1.37e-16 |
| rs3808976 | G | A | 0.00981886 | 0.00170108 | 7.84e-09 |
| rs99780 | T | C | 0.020203 | 0.00143553 | 5.73e-45 |
| rs35169799 | T | C | 0.0247241 | 0.00283283 | 2.61e-18 |
| rs678614 | A | C | 0.00935072 | 0.00153217 | 1.04e-09 |
| rs2302883 | C | T | 0.00886478 | 0.00162296 | 4.71e-08 |
| rs187217942 | A | G | 0.031159 | 0.00540272 | 8.06e-09 |
| rs17119701 | G | A | 0.0370675 | 0.00375036 | 4.94e-23 |
| rs61362984 | G | A | -0.0139461 | 0.00142207 | 1.06e-22 |
| rs61904855 | A | C | 0.0233784 | 0.00409645 | 1.15e-08 |
| rs11216122 | T | G | -0.0181908 | 0.00310342 | 4.59e-09 |
| rs7930786 | C | G | 0.124688 | 0.00278666 | 1e-200 |
| rs56225305 | A | G | 0.108415 | 0.00278601 | 1e-200 |
| rs2075294 | T | G | 0.0388118 | 0.00579905 | 2.19e-11 |
| rs75919952 | T | C | -0.046805 | 0.0031699 | 2.56e-49 |
| rs11600380 | C | T | -0.03673 | 0.00254055 | 2.34e-47 |
| rs5110 | A | C | -0.0185398 | 0.00248476 | 8.59e-14 |
| rs12721078 | A | C | -0.0322797 | 0.0039376 | 2.46e-16 |
| rs71480323 | A | G | -0.0195603 | 0.00211444 | 2.24e-20 |
| rs11216236 | T | C | 0.0240392 | 0.00342403 | 2.21e-12 |
| rs187929675 | T | C | -0.0767868 | 0.00606752 | 1.07e-36 |
| rs11045171 | G | A | -0.0116623 | 0.00173721 | 1.91e-11 |
| rs67981690 | G | A | 0.0148562 | 0.00207488 | 8.09e-13 |
| rs10783828 | A | G | 0.00903509 | 0.00147493 | 9.04e-10 |
| rs7296326 | C | T | -0.0118561 | 0.00217251 | 4.84e-08 |
| rs1585705 | C | A | 0.00876614 | 0.001496 | 4.64e-09 |
| rs10861679 | C | T | 0.00935439 | 0.00150663 | 5.35e-10 |
| rs1882491 | C | T | -0.0134975 | 0.00148108 | 8.04e-20 |
| rs1716407 | G | A | -0.0150613 | 0.00139861 | 4.9e-27 |
| rs7140110 | C | T | 0.0143695 | 0.00150803 | 1.61e-21 |
| rs112740904 | G | T | -0.0149023 | 0.00195835 | 2.76e-14 |
| rs12885801 | A | C | 0.00908515 | 0.00162151 | 2.11e-08 |
| rs34820917 | A | G | -0.0157999 | 0.00285011 | 2.97e-08 |
| rs35477346 | C | T | 0.00929684 | 0.00149721 | 5.32e-10 |
| rs139974673 | C | T | 0.0717689 | 0.00443008 | 5.34e-59 |
| rs72739147 | T | A | -0.0121232 | 0.00206079 | 4.04e-09 |
| rs1532085 | A | G | 0.0180035 | 0.0014104 | 2.64e-37 |
| rs261334 | G | C | 0.0261448 | 0.00167767 | 9.88e-55 |
| rs11636087 | C | T | 0.0116533 | 0.00154515 | 4.65e-14 |
| rs8028620 | C | T | -0.00897866 | 0.00137468 | 6.53e-11 |
| rs7175132 | G | A | -0.00811532 | 0.00141367 | 9.44e-09 |
| rs8025505 | T | C | 0.00964703 | 0.00158419 | 1.13e-09 |
| rs9935836 | C | A | 0.00988172 | 0.00177279 | 2.49e-08 |
| rs11075253 | A | C | -0.0141141 | 0.00150196 | 5.65e-21 |
| rs12446515 | T | C | -0.0187602 | 0.00147498 | 4.76e-37 |
| rs5880 | C | G | 0.0221143 | 0.00298074 | 1.18e-13 |
| rs12934528 | C | T | 0.0135148 | 0.00195818 | 5.15e-12 |
| rs2925979 | T | C | 0.0154375 | 0.00149811 | 6.79e-25 |
| rs11651957 | A | G | 0.0186479 | 0.00293615 | 2.14e-10 |
| rs12937081 | G | A | 0.0108836 | 0.00189096 | 8.64e-09 |
| rs72836561 | T | C | 0.0682178 | 0.00389919 | 1.69e-68 |
| rs231539 | T | C | 0.0130645 | 0.00188077 | 3.76e-12 |
| rs11657238 | A | G | -0.00784868 | 0.00138378 | 1.41e-08 |
| rs1801689 | C | A | -0.0293183 | 0.00408097 | 6.78e-13 |
| rs77244849 | C | T | -0.00875261 | 0.00148835 | 4.09e-09 |
| rs9891030 | A | G | 0.00993826 | 0.00159406 | 4.54e-10 |
| rs71352934 | C | A | -0.0163114 | 0.00273609 | 2.5e-09 |
| rs8092347 | G | A | 0.00812063 | 0.00141115 | 8.69e-09 |
| rs197156 | G | A | -0.00925016 | 0.00145381 | 1.99e-10 |
| rs1035941 | A | G | 0.0110148 | 0.00153309 | 6.75e-13 |
| rs4804413 | T | C | 0.00948499 | 0.00138516 | 7.53e-12 |
| rs116843064 | A | G | -0.108878 | 0.00491157 | 8.79e-109 |
| rs57192995 | C | G | -0.019903 | 0.00302387 | 4.65e-11 |
| rs58542926 | T | C | -0.0520456 | 0.00257716 | 1.26e-90 |
| rs188247550 | T | C | -0.0644712 | 0.00642107 | 1.02e-23 |
| rs62102718 | T | A | 0.0115887 | 0.00152691 | 3.22e-14 |
| rs58895965 | A | C | 0.0127238 | 0.00180395 | 1.75e-12 |
| rs541012177 | T | G | 0.0242543 | 0.00353606 | 6.94e-12 |
| rs41290102 | T | C | -0.0331177 | 0.00588779 | 1.86e-08 |
| rs419925 | C | G | -0.0130424 | 0.00149941 | 3.39e-18 |
| rs483082 | T | G | 0.0446753 | 0.00161686 | 8.09e-168 |
| rs79429216 | A | G | 0.0378212 | 0.0062985 | 1.92e-09 |
| rs146390218 | G | A | 0.0355235 | 0.00435052 | 3.22e-16 |
| rs62132802 | T | C | -0.00911832 | 0.00150245 | 1.29e-09 |
| rs12610709 | A | G | 0.013994 | 0.00183258 | 2.24e-14 |
| rs2207132 | A | G | 0.0283225 | 0.00387832 | 2.83e-13 |
| rs2250900 | T | C | 0.00899617 | 0.00163964 | 4.1e-08 |
| rs6073958 | C | T | 0.0274094 | 0.00172428 | 7.14e-57 |
| rs4812995 | C | T | 0.0091331 | 0.00161739 | 1.64e-08 |
| rs6066138 | A | G | -0.00850564 | 0.00152344 | 2.36e-08 |
| rs6090040 | A | C | 0.0089174 | 0.00138822 | 1.33e-10 |
| rs2277844 | G | A | -0.00908433 | 0.00138503 | 5.43e-11 |

**Supplementary Table 3.** Sensitivity analysis of the MR analysis results of exposures and outcomes

| **Exposure** | **Outcome** | **Heterogeneity test** | **Pleiotropy test** | **MR-PRESSO** | |
| --- | --- | --- | --- | --- | --- |
|  |  | Cochran’s Q test (*P* value) | Egger intercept (*P* value) | Distortion test | Global  test |
|  |  | IVW | MR-egger | Outliers | *P* Value |
| TyG | AD^*^ | 0.069 | 0.248 | NA | 0.066 |
|  | AA^*^ | ＜0.001 | 0.151 | NA | ＜0.001 |
|  | AD^#^ | 0.618 | 0.864 | NA | 0.596 |
|  | AA^#^ | 0.131 | 0,135 | NA | 0.148 |

**Abbreviation:** IVW, inverse-variance weighted; MR**,** Mendelian randomization.* From the FinnGen GWAS, ^#^ From the GWAS Catalog database.

**Supplementary Table 4**. Logistic Regression Results from Central Chi Fuwai Hospital Cohort

| Model | Covariates Adjusted | Odds Ratio (OR) | 95% CI | P-value | VIF-issue |
| --- | --- | --- | --- | --- | --- |
| 1 | Unadjusted | 1.325 | 1.236–1.421 | 2.49E-15 | NO |
| 2 | Adjusted for Age and Sex | 1.087 | 1.001–1.179 | 0.0467 | NO |
| 3 | Adjusted for Age, Sex, and hypertension | 1.140 | 1.051–1.237 | 0.00158 | NO |
| 4 | Model 3 + LDL-C | 1.199 | 1.108–1.296 | 5.75E-06 | NO |
| 5 | Model 3 + CHOL | 1.660 | 1.519–1.814 | <2E-16 | NO |
| 6 | Model 3 + Lipid Principal Component | 1.401 | 1.290–1.521 | 1.11E-15 | NO |

**Abbreviation:** CHOL: cholesterol. CI: Confidence interval.LDL-c: low density lipoprotein. OR: Odds ratio. VIF: Variance inflation factor.
